# Progression from *Candida auris* Colonization Screening to Clinical Case Status, United States, 2016–2023

**DOI:** 10.3201/eid3108.250315

**Published:** 2025-08

**Authors:** Anna D. Baker, Jeremy A.W. Gold, Kaitlin Forsberg, Sophie Jones, Meghan M. Lyman

**Affiliations:** Centers for Disease Control and Prevention, Atlanta, Georgia, USA

**Keywords:** *Candida auris*, candidemia, fungi, fungal drug resistance, antimicrobial resistance, cross infection, infection control, United States

## Abstract

During 2016–2023, among 21,195 US patients who tested positive for *Candida auris* colonization, 6.9% were subsequently found to have a positive clinical specimen (2.8% from blood). Strategies are needed to prevent invasive *C. auris* infections among patients with colonization (e.g., through patient decolonization).

*Candida auris*, an emerging, frequently antifungal-resistant yeast, can colonize patients asymptomatically and persist on skin for months to years without causing infection (*1*–*3*). Patients colonized with *C. auris* can progress to having invasive infections, which are associated with crude mortality rates of 30%–72% (*4*,*5*). Because *C. auris* spreads easily in healthcare settings, the Centers for Disease Control and Prevention (CDC) recommends colonization screening for patients with high-risk healthcare exposures (e.g., recent stay in a long-term acute-care hospital [LTACH] or ventilator-capable skilled nursing facility [SNF]) and those with an epidemiologic link to a patient with *C. auris* (*2*,*6*) (https://www.cdc.gov/candida-auris/hcp/screening-hcp).

Data characterizing the progression from *C. auris* colonization to invasive disease are limited but might help guide public health surveillance, prevention, and treatment efforts. We analyzed US national case-based surveillance data to characterize patients with positive *C. auris* screening results who were subsequently found to have a positive clinical specimen.

## The Study

*C. auris* is a nationally notifiable condition, but reporting mandates vary across states and jurisdictions. State and jurisdictional health departments report *C. auris* screening and clinical cases to CDC. Screening cases were defined as a positive *C. auris* laboratory result from a swab sample (usually composite axilla/groin) collected to test for colonization. Clinical cases were defined as a positive *C. auris* laboratory result from a clinical specimen collected to determine the cause and treatment for infection in a patient. Clinical cases might involve body sites typically associated with invasive infection (e.g., blood) or those that potentially reflect colonization (e.g., urine) (https://ndc.services.cdc.gov/case-definitions/candida-auris-2023). Screening and clinical case data included information on patient age and sex, as well as date and facility type of specimen collection. Facility location was grouped by Antimicrobial Resistance Laboratory Network region (n = 7) (https://www.cdc.gov/antimicrobial-resistance-laboratory-networks/php/about/domestic.html). We used a patient-level identifier to link each patient’s screening case with a clinical case, if one occurred. We considered patients with the corresponding clinical case >1 calendar day after a screening case to have a screening-to-clinical (StC) event. 

The analysis includes each patient’s screening case on the basis of their first positive screening result (StC and non-StC events) and clinical case on the basis of first positive clinical specimen (StC events only) during 2016–2023. We calculated total and annual percentages of patients with screening cases who had StC events and described available data on non-StC events and StC events, stratifying by StC event status and examining StC events by body site involved. We analyzed categorical data using χ^2^ tests and continuous data using Kruskal-Wallis rank-sum tests (α = 0.05).

**Figure 1:**
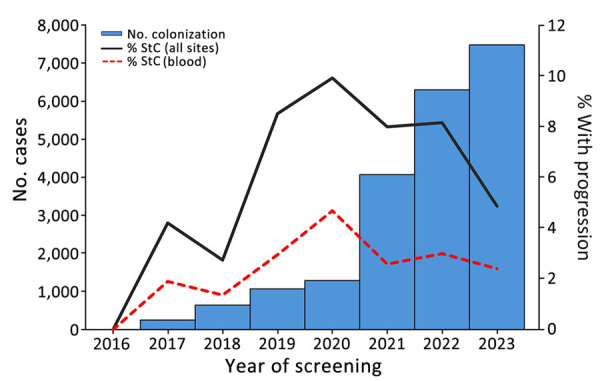
Number of patients with a *Candida auris* screening case and percentage who had progression to a clinical case, United States, 2016–2023. StC, screening-to-clinical.

During 2016–2023, a total of 36 of 40 reporting jurisdictions reported 21,195 patients who had a positive screening result; of those, 1,458 (6.9%) patients across 22 jurisdictions had an StC event (2.8% blood, 4.1% nonblood) (Table 1). The number of patients with screening cases increased each year, and the percentage of those with an StC event increased from 0.0% (0/13) in 2016 to 9.9% (129/1,299) in 2020, then decreased to 4.9% (365/7,493) in 2023 (Figure 1).

**Table 1:** Characteristics of patients with *Candida auris* screening cases with and without progression to clinical case status, United States, 2016–2023*

Characteristic	All, N = 21,195	With clinical case, n = 1,458	Without clinical case, n = 19,737	p value†
Median age at collection of screening case specimen, y (IQR), n = 17,928	68 (58–76)	67 (59–76)	68 (58–77)	0.650
Age group at collection of screening case specimen, y, n = 17,928				0.113
<45	1,738	118 (6.8)	1,620 (93.2)	
45–54	1,778	105 (5.9)	1,673 (94.1)	
55–64	3,725	279 (7.5)	3,446 (92.5)	
65–74	5,239	368 (7.0)	4,871 (93.0)	
75–84	3,808	274 (7.2)	3,534 (92.8)	
>85	1,640	94 (5.7)	1,546 (94.3)	
Sex, n = 16,446				0.478
M	9,448	668 (7.1)	8,780 (92.9)	
F	6,998	515 (7.4)	6,483 (92.6)	
Antimicrobial Resistance Laboratory Network region of the facility of collection for screening case specimen‡				<0.001
West	6,617	898 (13.6)	5,719 (86.4)	
Midwest	4,264	96 (2.3)	4,168 (97.7)	
Southeast	4,235	56 (1.3)	4,179 (98.7)	
Northeast	3,570	302 (8.5)	3,268 (91.5)	
Mid-Atlantic	1,484	64 (4.3)	1,420 (95.7)	
Mountain	977	42 (4.3)	935 (95.7)	
Central	48	0	48 (100.0)	
Facility type of screening case specimen collection, n = 17,357				<0.001
Long-term acute care hospital	8,716	907 (10.4)	7,809 (89.6)	
Acute care hospital	5,033	299 (5.9)	4,734 (94.1)	
Ventilator-equipped skilled nursing facility	2,912	150 (5.2)	2,762 (94.8)	
Skilled nursing facility	490	13 (2.7)	477 (97.3)	
Other¶	206	4 (1.9)	202 (98.1)	

*Values are no. (%) except as indicated. †p values were calculated using χ^2^ tests to compare characteristics of patients with a *C. auris* screening case who had (vs. did not have) progression to a *C. auris* clinical case. ‡From https://www.cdc.gov/antimicrobial-resistance-laboratory-networks/php/about/domestic.html. Data from 2023 were unavailable from 1 state in the Central region. ¶Includes those listed as other (n = 193), inpatient rehabilitation (n = 12), and outpatient (n = 1).

Among patients with screening cases (n = 21,195), the median age was 68 (interquartile range [IQR] 58–76) years; of those with known sex (n = 16,446), 9,448 (57.4%) were men and 6,998 (42.6%) were women (Table 1). The most common regions of screening case specimen collection were the West (31.2%, n = 6,617), Midwest (20.1%, n = 4,264), and Southeast (20.0%, n = 4,235) and the most common facility types among those with known facility type (n = 17,357) were LTACH (50.2%, n = 8,716), acute care hospital (ACH) (29.0%, n = 5,033), and ventilator-capable SNF (16.8%, n = 2,912). StC event frequency was similar by age (p = 0.650) and sex (p = 0.478) and varied by region (p<0.001), and facility type (p<0.001). StC event frequency was similar between women (7.4%) and men (7.1%) and was greatest among patients with screening specimens collected in the West (13.6%), Northeast (8.5%), Mid-Atlantic (4.3%), or Mountain (4.3%) regions. StC events were most frequent for patients with screening specimens collected in LTACHs (10.4%), then ACHs (5.9%), ventilator-capable SNFs (5.2%), non–ventilator-equipped SNFs (2.7%), and other facility types (1.9%).

**Figure 2:**
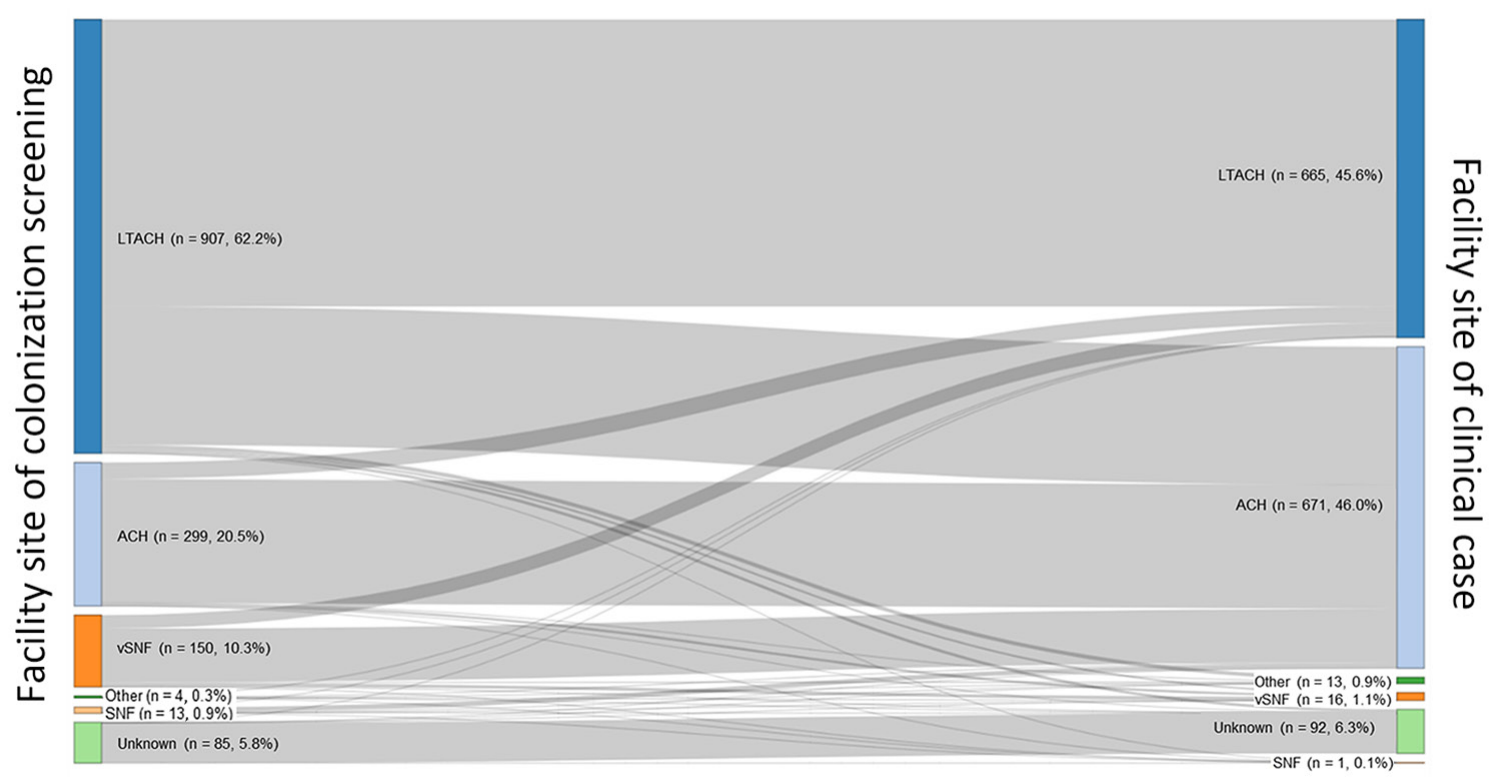
Facility type of specimen collection for patients with *Candida auris* screening cases in whom clinical *C. auris* cases occurred, United States, 2016–2023. Sankey diagram made in RStudio (https://www.rstudio.com). ACH, acute care hospital; LTACH, long-term acute-care hospital; SNF, skilled nursing facility (non–ventilator-equipped); vSNF, ventilator-equipped skilled nursing facility.

Among StC events (n = 1,458), blood (40.1%, n = 584) and urine (26.8%, n = 391) were most common (Table 2); the distribution of affected body sites was generally similar across years (Appendix Figure). Body sites of clinical cases varied by age (p = 0.023), sex (p<0.001), region (p<0.001), and time from screening case to clinical case specimen collection (p = 0.001) (Table 2). Among women, blood specimens were approximately twice as common as urine (42.6% vs. 21.5%), whereas among men, the percentage was similar (32.0% vs. 32.2%). Blood specimens constituted most StC events in the Southeast (58.9%), Northeast (58.6%), and Mid-Atlantic (57.8%) regions but less than half of specimens in other regions. The median number of days from initial screening case specimen to clinical specimen was longest for blood (58, IQR 22–130, range 1–1,309 days) and shortest for respiratory (33, IQR 17–74, range 1–1,240 days) and other (28, IQR 14–77, range 1–745 days) specimen types. The most common facility types of initial screening case detection were LTACHs (62.2%) and ACHs (20.5%) (Figure 2). Regardless of the facility type where the screening case was detected, most StC events were detected in an LTACH (45.6%) or ACH (46.0%).

**Table 2:** Patients with *Candida auris* screening cases with progression to clinical case status, by body site of clinical case detection, United States, 2016–2023*

Characteristic	All, N = 1,458	Blood, n = 584	Urine, n = 391	Respiratory, n = 233	Wound, n = 168	Other, n = 82†	p value‡
Median age at collection of screening case specimen, y (IQR), n = 1,238	67 (59–76)	68 (60–76)	68 (58–77)	68 (60–77)	67 (61–75)	64 (50–71)	0.023
Age group at collection of screening case specimen, y, n = 1,238							0.027
<45	118	42 (35.6)	40 (33.9)	8 (6.8)	15 (12.7)	13 (11.0)	
45–54	105	35 (33.3)	27 (25.7)	21 (20.0)	11 (10.5)	11 (10.5)	
55–64	279	106 (38.0)	69 (24.7)	55 (19.7)	38 (13.6)	11 (3.9)	
65–74	368	134 (36.4)	97 (26.4)	68 (18.5)	48 (13.0)	21 (5.7)	
75–84	274	106 (38.7)	83 (30.3)	43 (15.7)	31 (11.3)	11 (4.0)	
>85	94	33 (35.1)	26 (27.7)	22 (23.4)	12 (12.8)	1 (1.1)	
Sex, n = 1,183							<0.001
M	671	215 (32.0)	216 (32.2)	124 (18.5)	76 (11.3)	40 (6.0)	
F	512	218 (42.6)	110 (21.5)	84 (16.4)	73 (14.3)	27 (5.3)	
Antimicrobial Resistance Laboratory Network region of the facility of specimen collection for clinical case§							<0.001
West	898	306 (34.1)	252 (28.1)	173 (19.3)	124 (13.8)	43 (4.8)	
Midwest	96	24 (25.0)	36 (37.5)	22 (22.9)	8 (8.3)	6 (6.3)	
Southeast	56	33 (58.9)	10 (17.9)	7 (12.5)	3 (5.4)	3 (5.4)	
Northeast	302	177 (58.6)	68 (22.5)	25 (8.3)	20 (6.6)	12 (4.0)	
Mid-Atlantic	64	37 (57.8)	11 (17.2)	3 (4.7)	4 (6.3)	9 (14.1)	
Mountain	42	7 (16.7)	14 (33.3)	3 (7.1)	9 (21.4)	9 (21.4)	
Central¶	0	0 (NA)	0 (NA)	0 (NA)	0 (NA)	0 (NA)	
No. days from collection date of first positive screening to first clinical case specimen#							0.001
Median (IQR)	46 (19–108)	58 (22–130)	44 (20–120)	33 (17–74)	44 (17–91)	28 (14–77)	
Minimum–maximum	1–1,597	1–1,309	1–1,597	1–1,240	1–666	1–745	

*Values are no. (%) except as indicated. IQR, interquartile range; NA, not applicable. †Other specimen type included device (n = 14), fluid/drainage (n = 5), intraabdominal (n = 2), other (n = 60), and unknown (n = 1). ‡p values were calculated using χ^2 ^tests (categorical variables) or Kruskal-Wallis rank-sum test (continuous variables) to compare features of interest by body site. §https://www.cdc.gov/antimicrobial-resistance-laboratory-networks/php/about/domestic.html. Data from 2023 were unavailable from 1 state in the Central region. ¶Row excluded from p value calculations because no screening-to-clinical cases were reported in the Central region. #Data missing for 1 patient with a clinical case involving a wound.

## Conclusions

This analysis of national *C. auris* case data revealed that, among 21,195 patients who tested positive for *C. auris* on a colonization screening swab during 2016–2023, a clinical case subsequently occurred in 6.9% (2.8% involving blood); more than half of clinical cases involving blood were detected 2 months after screening case detection. This finding is comparable with a smaller New York state study in which a *C. auris* bloodstream infection occurred in 7/187 (3.7%) colonized patients (median time from screening case testing to infection 86 days) (*7*). 

The percentage of patients with an StC event peaked in 2020 then declined, potentially because of improved infection prevention and control efforts or increased screening after COVID-19–related resource strains resolved. The volume of screening cases and frequency of clinical cases was greatest in the West, but the region had a relatively low percentage of clinical cases involving blood; that finding might reflect regional differences in case reporting and in testing practices for *C. auris* in noninvasive body sites (*8*). Most StC events were identified in LTACHs and ACHs, underscoring the continued need for focused screening, enhanced surveillance, and efforts to improve infection prevention and control implementation in these settings.

For several reasons, we suspect that our study underestimates the actual percentage of patients with *C. auris* colonization who progress to having a clinical case. StC events could have been missed because of missed screening opportunities, the insensitivity of culture (*9*), treating clinical laboratories that might not routinely distinguish *C. auris* from other *Candida* species for nonsterile specimen types (*10*), and the fact that US *C. auris* data from 2024 are not finalized, meaning some patients might not have had sufficient lead time for clinical cases to occur. In addition, for clinical cases, we lacked data on previous negative screening results, the differentiation between infection and colonization, and underlying patient conditions.

Overall, our study highlights the potential for *C. auri*s infections, particularly candidemia, among patients colonized with *C. auris.* Rigorous infection prevention and control remain necessary to prevent the spread of *C. auris* and subsequent clinical infections. Further studies could investigate risk factors and strategies to prevent invasive *C. auris* infections among patients with colonization (e.g., through patient decolonization).

Appendix Additional information about progression from *Candida auris* colonization screening to clinical case status, United States, 2016–2023
