# Supplementary material for: Progression from Candida auris Colonization Screening to Clinical Case Status, United States, 2016–2023
Source: Emerg Infect Dis. 2025 Aug;31(8):1613–7. doi: 10.3201/eid3108.250315 (PMC12309774; doi:10.3201/eid3108.250315)
Supplement: Appendix — Additional information about progression from Candida auris colonization screening to clinical case status, United States, 2016–2023 [file 25-0315-Techapp-s1.pdf]

Article DOI: https://doi.org/10.3201/eid3108.250315

EID cannot ensure accessibility for supplementary materials supplied by authors. Readers who have difficulty accessing supplementary content should contact the authors for assistance.

Progression from *Candida auris* Colonization Screening to Clinical Case Status, United States, 2016–2023

Appendix

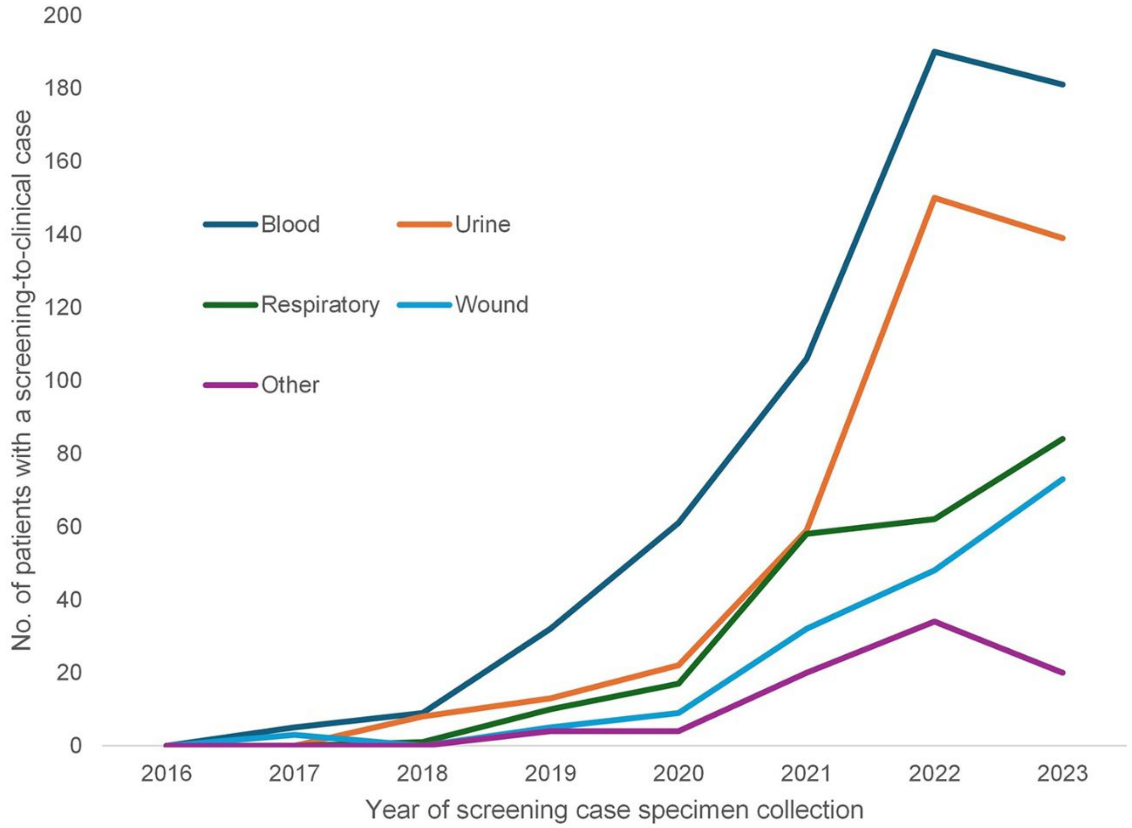

Appendix Figure. Number of patients with a Candida auris screening case who had progression to a clinical case by body site of clinical case, United States, 2016–2023
